# Revision of the genus *Coccidula* Kugelann (Coleoptera, Coccinellidae)

**DOI:** 10.3897/zookeys.1043.65829

**Published:** 2021-06-11

**Authors:** Karol Szawaryn, Oldřich Nedvěd, Amir Biranvand, Tomasz Czerwiński, Romain Nattier

**Affiliations:** 1 Museum and Institute of Zoology, Polish Academy of Sciences, Wilcza 64, 00-679 Warszawa, Poland Museum and Institute of Zoology, Polish Academy of Sciences Warszawa Poland; 2 Faculty of Science, University of South Bohemia, Branišovská 1760, CZ-37005 České Budějovice, Czech Republic University of South Bohemia České Budějovice Czech Republic; 3 Czech Academy of Sciences, Biology Centre, Institute of Entomology, České Budějovice, Czech Republic Czech Academy of Sciences, Biology Centre, Institute of Entomology Česk&eacute; Budějovice Czech Republic; 4 Department of Entomology, College of Agricultural Sciences, Shiraz Branch, Islamic Azad University, Shiraz, Iran Islamic Azad University Shiraz Iran; 5 Institut de Systématique, Evolution et Biodiversité (ISYEB), Muséum national d'Histoire naturelle, CNRS, Sorbonne Université, EPHE, Université des Antilles, 57 rue Cuvier, CP 50, 75231, Paris Cedex 05, France Sorbonne Université Paris France

**Keywords:** Coccinelloidea, ladybirds, morphological revision, new synonym, taxonomy

## Abstract

The genus *Coccidula* Kugelann includes five species distributed in the Holarctic, with one species in North America and four in Palearctic region. *Coccidula* belongs to the tribe Coccidulini which historically was treated as a separate subfamily within ladybird beetles, but recent studies confirmed its placement as a tribe within the broadly defined subfamily Coccinellinae. All species are revised and a **new synonymy** of *Lithophilus naviauxi* Duverger with *C. litophiloides* Reitter is proposed. Light and electron microscopy pictures support morphological descriptions. An identification key to all species is also provided.

## Introduction

The classification of ladybird beetles (Coccinellidae) has changed dynamically in the last decade mainly due to molecular approaches. Although several studies have been conducted at the family level, none of them gave robust classification of the family (Seago et al. 2011; Robertson et al. 2015). Historically, the family was divided into six or seven subfamilies (Sasaji 1968; Kovář 1996) but more recent treatments based on morphology and molecules support just two, Microweiseinae and Coccinellinae (Ślipiński 2007; Seago et al. 2011; Robertson et al. 2015). However, recent analysis of a large molecular dataset (Che et al. 2021) revealed the existence of the third monotypic subfamily Monocoryninae.

The genus *Coccidula* Kugelann, 1798 was traditionally placed in the subfamily Coccidulinae (Sasaji 1968; Kovář 1996), nonetheless, in the new classification of ladybirds it was proposed to be one of the tribes (Coccidulini) within the broadly defined subfamily Coccinellinae. Seago et al. (2011) synonymized this tribe with Scymnini, however, after the analyses by Robertson et al. (2015) and Che et al. (2021) both are once again treated as independent tribes. Coccidulini are one of the most problematic groups of ladybirds as in the traditional classification they contain numerous genera with just superficial external similarity based mainly on hairy body surface and relatively long antennae. Consequently, in all molecular analyses they do not form a monophyletic group (Seago et al. 2011; Robertson et al. 2015; Che et al. 2021). The tribe is distributed worldwide with moderate diversity in the Palearctic (Kovář 2007) and African regions (Fürsch 2007; Tomaszewska 2010), rich in South America (Gordon 1994), but the most diverse fauna occurs in South Asia, Australia and neighboring regions (e.g., Ślipiński 2007; Poorani and Ślipiński 2009; Tomaszewska 2010; Tomaszewska and Ślipiński 2011; Szawaryn and Leschen 2019). The largest and most widely distributed is the genus *Rhyzobius* Stephens, 1831 with more than 100 recognized species (Tomaszewska 2010; Czerwiński et al. 2020) and numerous undescribed species mainly from New Guinea. Interestingly it is also the only genus of Coccidulini with known fossil representatives from the Eocene period discovered in Oise (Kirejtshuk and Nel 2012) and Baltic ambers (Szawaryn and Tomaszewska 2020).

*Coccidula* is a small genus distributed in the Holarctic, with one species in North America and four in Eurasia. Historically numerous species and varieties have been described based mostly on differences in color pattern, but most of them were subsequently synonymized when genitalia were examined. Gordon (1985) revised the North American species; however, the Palearctic species have not been revised until now. As Coccidulini has never been a subject of morphological cladistic analysis there is no hypothesis about its internal relationship available. However, based on recent molecular analyses (Che et al. 2021), *C. scutellata* (Herbst, 1783) and *Rhyzobius litura* (Fabricius, 1787) group in a single clade with the African genus *Epipleuria* Fürsch, 2001 and African species of *Rhyzobius*.

The European species are usually found in wetlands and water banks in low and middle elevations (Bielawski 1959). They live on herbaceous emergent grassy vegetation such as reeds, feeding on aphids such as *Hyalopterus pruni* (Hemiptera: Aphididae). *Coccidula rufa* is sometimes contrastingly reported also from dry sand dunes and in Finland from cereal fields (Clayhills and Markkula 1974). High prevalence (60–80%) of endosymbiotic bacteria *Rickettsia* and *Wolbachia* was reported from Germany (Weinert et al. 2007). *Coccidula rufa* is univoltine – mating and egg-laying occur in the spring and summer, eggs are laid in batches on reed stems and foliage, larvae develop through the spring and summer, and a new generation of adults emerges in July. All species may be locally and temporally abundant.

The tribe Coccidulini needs comprehensive revision. In the current work we present a morphological revision of all currently known species of *Coccidula*, the type genus for the tribe. This revision is a first step to understand the morphological diversity of the tribe and may lead to further phylogenetic studies.

## Material and methods

Material used of this study is deposited in the following collections:

**AJC** Andrzej Jadwiszczak Collection, Poland;

**ASC** Alexander Slutsky Collection, Kharkov, Ukraine;

**HNHM**Hungarian Natural History Museum, Budapest, Hungary;

**NMP**National Museum Prague, Czech Republic;

**MIZ** Museum and Institute of Zoology, Warsaw, Poland;

**MNHN**Muséum national d’histoire naturelle, Paris, France;

**USB**University of South Bohemia, České Budějovice, Czech Republic.

Genitalia were dissected, cleared in a 10% KOH solution, washed in water, and placed in glycerol on slides for further study. Female genitalia were stained with chlorazol black. Measurements were recorded as follows: TL – total body length from apical margin of clypeus to apex of elytra; PL – pronotal length from the middle of anterior margin to the middle of the posterior margin; PW – pronotal width across widest part; EL – elytral length along suture including scutellum; EW – elytral width across both elytra at the widest part. Colour images were taken using either a stereo microscope Leica MZ 16 with a digital camera IC 3D; final images were produced using Helicon Focus 5.0X64 and Adobe Photoshop CS6 software, or a stereo microscope Nikon SMZ 1500 with Lumenera digital camera and QuickPhoto software, composite images with deep focus were generated using Zerene Stacker. The SEM photographs were taken in the Laboratory of Scanning Microscopy, MIZ (Warsaw), using a scanning electron microscope HITACHI S-3400N under low vacuum conditions and on JEOL JSM-7401F in Biology Centre CAS (České Budějovice). Terminology used for morphology follows Ślipiński (2007) and Lawrence et al. (2011). In this paper, we follow the classification proposed by Che et al. (2021).

## Taxonomy

### Family Coccinellidae Latreille, 1807

**Subfamily Coccinellinae Latreille, 1807**

#### Tribe Coccidulini Mulsant, 1846

##### Coccidula

Taxon classificationAnimaliaColeopteraCoccinellidae

Kugelann, 1798

1336C156-E005-5A7D-8D59-839CE87A724A

Coccidula Kugelann, 1798: 421. Type species: Chrysomela scutellata Herbst, 1783, by subsequent designation by Crotch 1874.
Strongylus Panzer, 1813: 114.
Cacidula Dejean, 1821: 132. Type species: Chrysomela pectoralis Fabricius, 1792 (=Dermestes rufus Herbst, 1783).
Cacicula Stephens, 1831: 397.

###### Diagnosis.

Representatives of the genus *Coccidula* with its general body shape may resemble *Tetrabrachys* Kapur, however, it can be separated based on the structure of the tarsi which are tetramerous in both genera but in *Coccidula* the first tarsomere is sub-triangularly broadened apically and the second is elongate and distinctly lobbed, while in *Tetrabrachys* both the first and second are narrow, elongate and without lobes. Moreover, in *Tetrabrachys* the apical maxillary palpomere is widely securiform, and beetles are brachypterous, while in *Coccidula* the apical maxillary palpomere is only slightly widened and the second pair of wings is functional. *Coccidula* is also externally similar to European species of *Rhyzobius* but it can be separated based on the following characters: body almost parallel sided, elytra covered with punctures of two sizes, larger punctures arranged in nine rows (in *C. litophiloides* some of them are reduced), base of the pronotum not bordered, while in *Rhyzobius* the lateral body outline is broadly rounded, the elytra are covered with single sized, randomly arranged punctures, and base of the pronotum with distinct bordering line.

###### Description.

**Figure 1.**
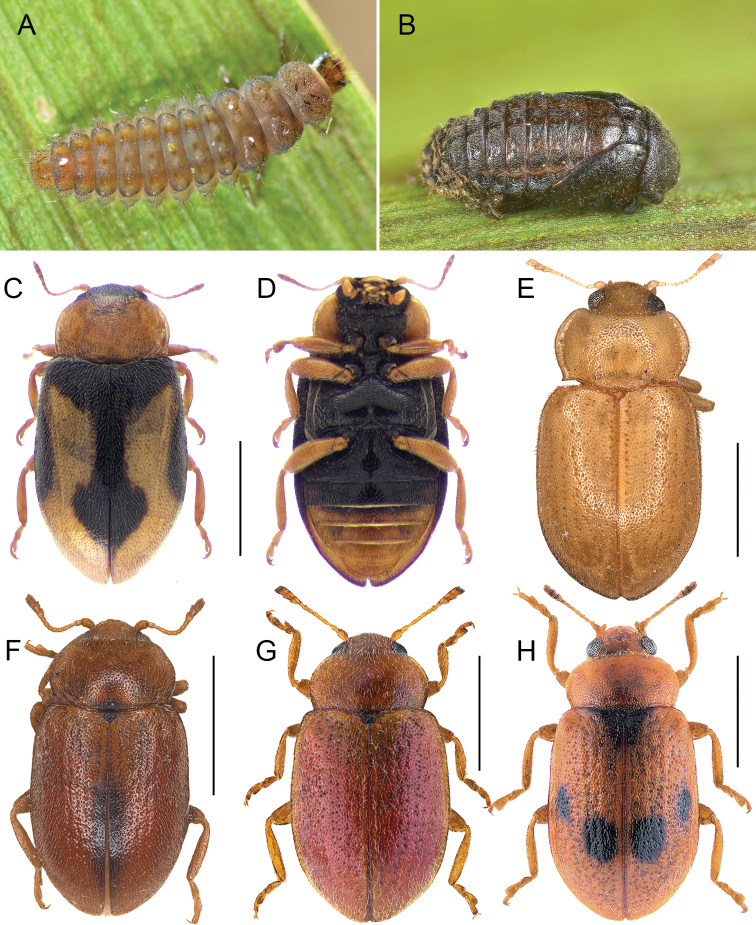
Immature stages and habitus of adult species of *Coccidula* Kugelann **A***C. rufa* larva **B***C. rufa* pupa **C***C. lepida*, dorsal **D***C. lepida*, ventral **E***C. litophiloides***F***C. reitteri***G***C. rufa***H***C. scutellata***A, B** Gilles San Martin, Wikimedia Commons **C, D** Danny Haelewaters **G, H** Udo Schmidt. Scale bars: 1 mm (**C–H**).

Body elongate-oval, with sides parallel (Fig. 1C–H), body flattened in lateral view, convex in cross-section; dorsum covered with setiferous punctures of two sizes (Figs 7A, 9A), hairs directed forwards on pronotum, backwards on elytra.

**Figure 2.**
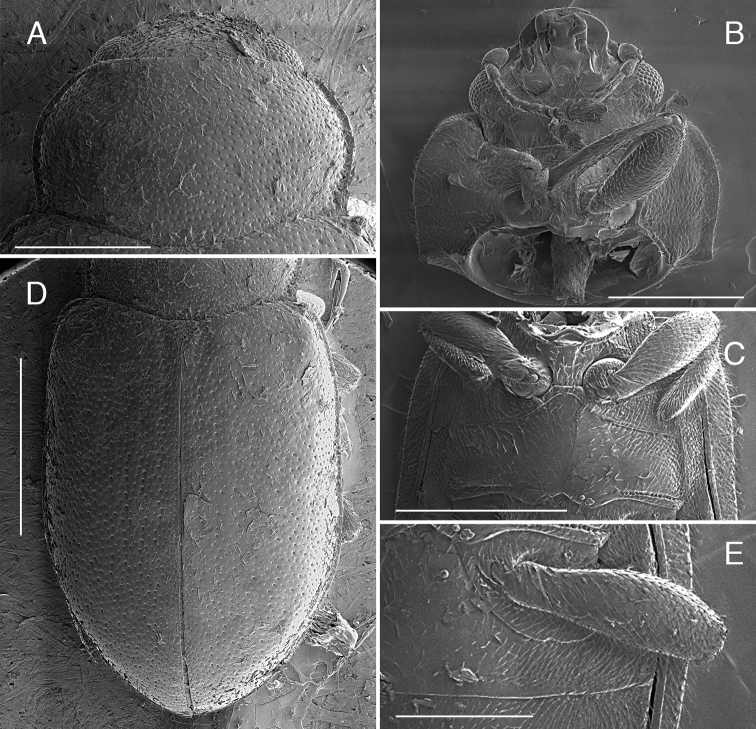
*Coccidula lepida* LeConte SEM illustrations **A** pronotum **B** head and prothorax, ventral **C** meso and metaventrite **D** elytra dorsal **E** ventrite 1. Scale bars: 500 µm **(A–C)**; 1 mm (**E, D**).

**Figure 3.**
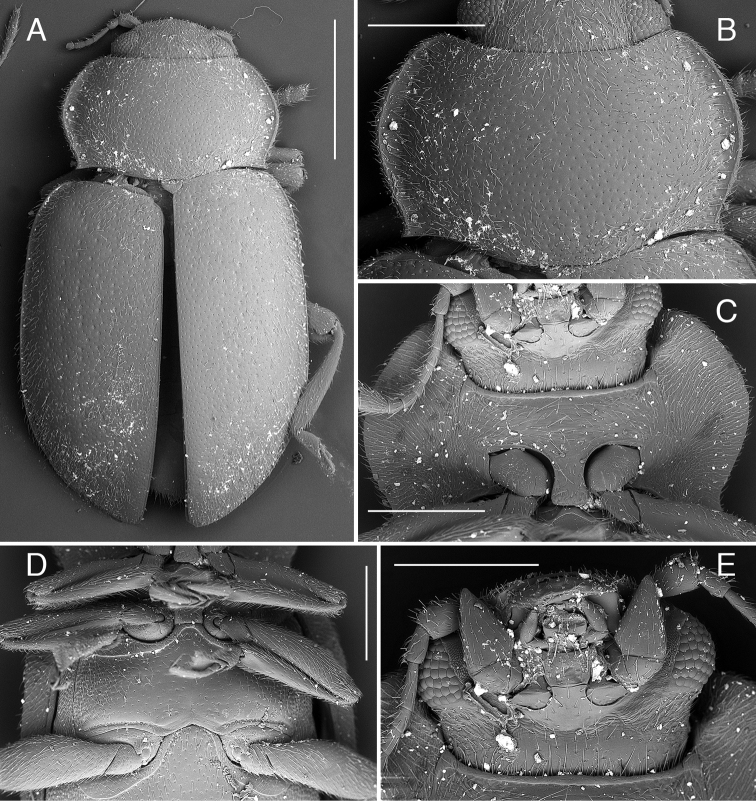
*Coccidula litophiloides* Reitter SEM illustrations, paratype HNHM**A** body, dorsal **B** pronotum **C** head and prothorax, ventral **D** meso and metaventrite **E** head ventral. Scale bars: 1 mm (**A**); 500 µm (**B–D**); 400 µm (**E**).

Head partially withdrawn into prothorax (Fig. 1C–H); ventral antennal grooves shallow and moderately long, extending to posterior border of an eye (Fig. 3E). Eyes prominent, coarsely facetted (7–8 ommatidia per eye width), ocular canthus distinct, about as long as 4–5 ommatidium diameters; interocular distance about 3× as eye diameter; interfacetal setae present only in basal part; temple behind eye distinctly longer than eye (Fig. 3E). Antennal insertion placed laterally, invisible from above, distance between antennal insertions about same as between eyes; frons around antennal insertions slightly expanded, covering antennal insertions, anterior tentorial pits placed ventrally below antennal insertions. Antennae (Figs 5A, E, 7A, D) longer than maximum head width including eyes, composed of 11 antennomeres (AN); scape simple, without projections, slightly curved; pedicel distinctly narrower than scape, elongate (1.5× longer than wide); AN 3–8 elongate (AN3 ≈ 3.5×; AN8 ≈ 1.3× longer than wide); AN 9–11 forming a loose, asymmetric club, ultimate AN truncate apically. Frontoclypeus short, transverse, anterior margin straight. Labrum entirely exposed, transverse, anterior margin straight. Mandibles asymmetric, bifid apically (Fig. 10G), molar part with basal tooth; prostheca distinct. Maxillary stipes (Figs 2B, 5C, 9C, 10H) with distinct groove for reception of maxillary palp in repose; palpomere 2 shorter than terminal (4^th^) one, slightly broadened apically; palpomere 3 about 2.3× shorter than terminal one, subtriangular; terminal palpomere slightly securiform; lacinia with stiff setae on outer margin in apical half, with several additional spurs on surface (Fig. 10H). Labial palps (Figs 3E, 9C) with 3 palpomeres, inserted ventrally on prementum; palpomere 1 very small, apical palpomere as long as and about as broad as penultimate; distance between palp insertions about 1.5–2× as its width. Prementum subquadrate, transverse apically. Mentum trapezoidal, broadest in anterior part, with horseshoe impression at base (Figs 7C, 9C). Submentum broad, transverse, with suture invisible.

Anterior margin of pronotum weakly, broadly emarginate (Figs 3B, 5B) with anterior corners broadly rounded; lateral margins with moderately (Figs 7B, 9B) to distinctly expanded lateral beads (Fig. 3B), distinctly margined; hind corners sharply pointed; hind margin not bordered. Prothoracic hypomeron smooth, without delimited foveae (Figs 3C, 7C). Prosternum in front of coxae about as long as longitudinal length of procoxal cavity; anterior margin straight or slightly emarginate with distinct border. Prosternal process about 0.4 times of coxal diameter, surface smooth (Fig. 3C) or with lateral carinae (Figs 7C, 9E). Procoxal cavity oval, distinctly bordered anteriorly.

Mesoventrite 1.3× longer than its width at the level of mid coxae (Figs 1D, 5D, 7D); mesal surface with deep emargination for receiving tip of prosternal process (Fig. 2C); anterior margin with completely raised border. Meso-metaventral process narrow (Figs 1D, 2C, 5D, 7E), about 0.5 times of mesocoxal diameter, junction slightly arcuate (Figs 2C, 3D, 5D, 7E, 9D), with suture visible. Metendosternite with stalk sub-quadrate, tendons long, separated by a distance of about width of stalk and situated closer to center (Fig. 10I). Scutellar shield pentagonal (Figs 7B, 9B). Elytra at base wider than pronotum, lateral margins clearly visible from above throughout (Figs 2D, 3A, 5A, 7A) (except *C. scutellata* where it is obscured in basal part, Fig. 9A), surface covered with punctures of double size, smaller irregularly distributed, larger punctures arranged in nine irregular longitudinal rows. Sutural stria absent. Elytral epipleuron narrow, incomplete, reaching base of ventrite 4 (Fig. 1D), with complete bordering line, epipleural foveae absent. Hind wings fully developed or missing (in *C. litophiloides*). Metaventral postcoxal lines roundly joined medially, complete laterally, straight or descending (Figs 2C, 3D, 5D, 7E, 9D). Metaventrite with discrimen visible in posterior 2/3.

Trochanters simple, subtriangular, without projection (figs 7E, 9D). Tibiae slightly expanded apically with one apical spur on forelegs, and two in mid and hind legs. Tarsi consisting of four tarsomeres, second tarsomere truncate apically; tarsal claws cleft apically (Fig. 9G) with single empodial seta present.

Abdomen in both sexes with 6 ventrites (Fig. 1D); ventrite 1 about as long as ventrites 2–4 combined, ventrite 2 longer than ventrite 3, ventrites 3–5 subequal in length. Abdominal postcoxal lines (Figs 7E, 9D) separate medially, recurved and complete, reaching anterior margin of ventrite, posteriorly reaching about half length of ventrite 1. Ventrite 5 in female posteriorly rounded (Fig. 7F), in male truncate (Fig. 9F). Ventrite 6 rounded in both sexes.

**Figure 4.**
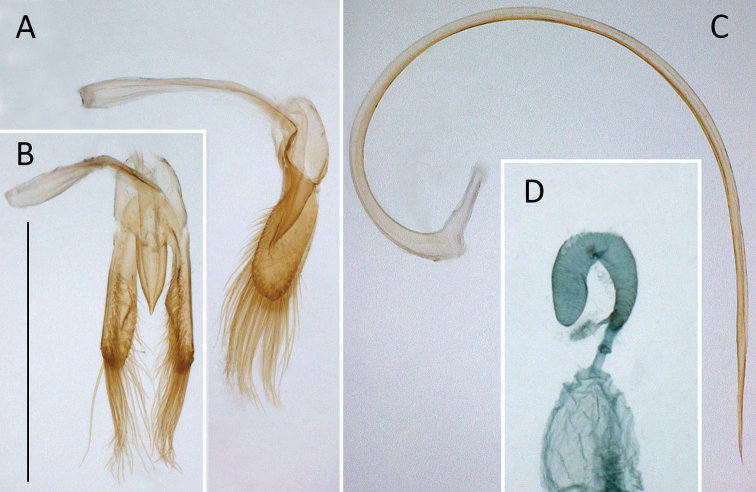
*Coccidula litophiloides* Reitter **A** tegmen, lateral **B** tegmen, inner **C** penis, lateral **D** spermatheca. Scale bar: 500 µm (**A–D**).

***Male terminalia*.** Tegmen (Figs 4A, B, 8A, B, 10A, B) symmetrical; parameres articulated with penis guide. Penis (Figs 4C, 8C, 10C) slender, pointed apically; penis capsule asymmetrical with outer arm reduced, inner arm well developed. Apodeme of male sternum IX simple, not broadened apically (Figs 8D, 10D). Tergite X broadly rounded, semicircular (Figs 8D, 10D).

**Figure 5.**
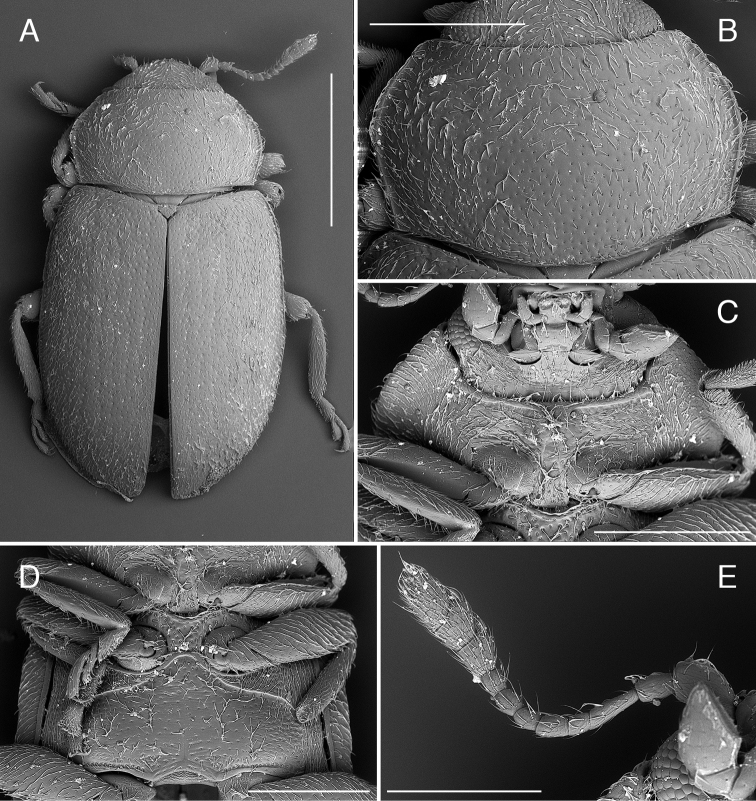
*Coccidula reitteri* Dodge SEM illustrations, HNHM**A** body, dorsal **B** pronotum **C** head and prothorax, ventral **D** meso and metaventrite **E** antenna. Scale bars: 1 mm (**A**); 500 µm (**B–D**); 300 µm (**E**).

***Female terminalia*.** Coxites (Figs 10F) distinctly elongate, subtriangular; styli small but visible, bearing several short setae; infundibulum absent (Figs 4D, 8E, 10E); sperm duct simple. Spermatheca (Figs 4D, 8E, 10E) worm-like, without clear ramus or nodulus; spermathecal accessory gland small, elongate. Proctiger elongate, rounded apically (Fig. 10F).

###### Immature stages.

Larva as in Fig. 1A, pupa as in Fig. 1B.

###### Distribution.

Holarctic: Asia, Europe, Africa (North), North America.

### Key to species of *Coccidula* Kugelann

**Table:** 

1	Head and epipleurae black (Fig. 1C, D); elytra with humeral area black. Nearctic	***C. lepida* LeConte**
–	Head and epipleurae testaceous; elytra with humeral area testaceous. Palaearctic	**2**
2	Pronotum with posterior corners pointed, with an angle much less than 90° (Figs 1G, 3B); pronotal lateral margins broadly explanate (Figs 1E, 3B); prosternal process without carinae (Fig. 3C); elytra with missing or reduced rows of large punctures 2 and 3 (counted from the suture)	***C. litophiloides* Reitter**
–	Pronotum with posterior corners not distinctly pointed, with an angle around 90° (Figs 1F–H, 7B, 9B); pronotal lateral margins moderately explanate (Figs 1G, 7B, 9B); prosternal process with distinct lateral carinae (Figs 7C, 9E); elytra with all rows of large punctures well visible	**3**
3	Prosternal process with lateral carinae very distinct, sinuate, roundly joined to the anterior prosternal margin (Fig. 9E); lateral elytral margins in basal part not visible from above (Fig. 9A); metaventral postcoxal lines narrowly separated on metaventral process (Fig. 9D); specimens entirely testaceous or with more than one black macula on elytra (Fig. 1H); penis guide about half length of parameres (Fig. 10A)	***C. scutellata* Herbst**
–	Prosternal process with lateral carinae straight, sometimes not joined together, extending to level of anterior border of procoxal cavity, not merged to anterior prosternal margin (Figs 5C, 7C); lateral elytral margins in basal part visible from above (Figs 5A, 7A); metaventral postcoxal lines joined on metaventral process (Figs 5D, 7E); specimens entirely testaceous to rufous (Fig. 1G) or with single elongate dark brown to black macula near the elytral suture around middle of elytra (Fig. 1F); penis guide longer than parameres (Figs 6B, 8A)	**4**
4	Body entirely rufous, sometimes with darker scutellar shield (Fig. 1G); penis guide distinctly curved in lateral view, parameres at base about as broad as in middle (Fig. 8B)	***C. rufa* (Herbst)**
–	Body testaceous with small transverse macula at base of pronotum just above scutellar shield, scutellar shield dark, elytra with single dark brown to black, longitudinal macula on elytral suture around middle of elytra (Fig. 1F); penis guide mildly curved in lateral view, parameres at base distinctly narrower than in middle (Figs 6A, C)	***C. reitteri* Dodge**

#### Coccidula lepida

Taxon classificationAnimaliaColeopteraCoccinellidae

LeConte, 1852

4CE4208F-E9C6-51F6-93F6-0F46591A20FB

Figs 1C, D , 2A–E

Coccidula lepida LeConte, 1852: 132.
Coccidula occidentalis Horn, 1895: 114.
Coccidula suturalis Weise, 1895: 132.

##### Material examined.

United States of America: America b., 82, coll. Růžička et Vokál, (1: NMP); Alaska, Mi.1249, Alaska Hwy., Dedman Lk., 6.–7.VII.1968, Campbell & Smetana (1: NMP); Vermont, Korschefsky det. (2: MIZ); Canada: SK, Harris Reservoir, Hwy 21, 10 km S, Maple Creek, April 19 2016, drift D. Larson (1 female: NMP). Type material not studied, deposited in Museum of Comparative Zoology, Cambridge, USA.

##### Diagnosis.

*Coccidula lepida* is the only Nearctic species of the genus, and is similar in many characters to *C. scutellata*, but with the head and epipleura black. In the typical form (*C. lepida* described by LeConte), the black elytral pattern resembles an extension of the five fused black maculae on the elytra of *C. scutellata*, with shoulders and epipleura black. Shoulder tubercle distinct, prototum relatively narrow. Male genitalia with penis guide much shorter than parameres. Detailed description of morphology including variability in pattern can be found in Gordon (1985: 656–659).

##### Description.

Length = 2.7–3.5 mm, BL/BW = 1.88–1.96, EL/BW = 1.40–1.42, PW/BW = 0.73.

Body elongate (Fig. 1C), slightly widening in posterior part. Head black. Elytra of typical form, light testaceous with black pattern covering scutellar shield and surrounding portion of elytra through shoulders to lateral margins, covering about 60% of its anterior part; pair of maculae in posterior 3/4 of elytra near suture; in western population fused and connected to scutellar shield over suture. Ventral side (Fig. 1D) black with hypomera and ventrites 3–6 testaceous.

Head and pronotum covered with uniform small setiferous punctures arranged irregularly. Pronotum transverse, broadly rounded laterally, with lateral margin glabrous; pronotum covered with dense setiferous punctures. Posterior pronotal corners not produced (Fig. 2A). Prosternum with anterior margin with bordering line complete. Prosternal process with complete lateral carinae, joined roundly and merged with anterior border of pronotum (Fig. 2B).

Scutellar shield pentagonal, covered with dense setiferous punctures. Elytra (Fig. 2D) covered with two types of punctures, small setiferous punctures irregularly distributed throughout the elytral surface, some of these punctures surrounded by larger depressed circles forming nine irregular longitudinal rows along the whole length of elytra. Shoulder tubercles distinct, but lateral elytral margin of elytra visible from above throughout. Mesoventrite (Fig. 2C) with anterior border interrupted in median part. Metaventrite (Fig. 2C) with postcoxal lines transverse in median part and then descending laterally, continuous on the metaventral process in median part; covered with setiferous punctures very sparsely distributed in central part of sclerite, densely setose in anterolateral parts, with a single row of large punctures below postcoxal lines and above metacoxae.

Abdominal postcoxal lines (Fig. 2E) complete, widely rounded, reaching about half of the length of the ventrite 1 measured below metacoxa. Ventrites covered with dense setiferous punctures.

***Male genitalia*.** Tegmen in inner view with penis guide subtriangular with pointed apex; short, about two times shorter than parameres. Parameres elongate elliptical, inner surface smooth, with long setae on the inner side and in apical margin. Penis simple with pointed apex. [see Gordon 1985: 657, fig. 539 a–d]

***Female genitalia*.** Sperm duct long, much longer than length of spermatheca. Spermatheca vermiform, broadest in basal part. [see Gordon 1985: 657, fig. 539e]

##### Type locality.

Vermont (USA).

##### Distribution.

North part of North America.

#### Coccidula litophiloides

Taxon classificationAnimaliaColeopteraCoccinellidae

Reitter, 1890

4CDB3460-0B84-58C1-9ED8-645728163B8E

Figs 1E , 3A–E , 4A–D

Coccidula litophiloides Reitter, 1890: 176
Lithophilus naviauxi Duverger, 1983: 83. syn. nov.

##### Material examined.

***Holotype*.** Azerbaijan, “Caucasus Araxesthal Leder Reitter/ Coll. Reitter/ Coccidula litophiloides 1890/ Holotypus 1890 Coccidula litophiloides Reitter”, male (HNHM) (Fig. 11C). ***Holotype*** of *L. naviauxi*, Iran, Vannae, 30-V-77, leg. M. Rapilly, female (MNHN). ***Paratypes*** of *C. litophiloides*. Data same as for the holotype, (7: HNHM). ***Paratypes*** of *L. naviauxi*: Iran, Daran, 9-VI-77, M. Rapilly leg. (2 females: MNHN) (Figs 11A, B). **Other material.** Armenia, Eczmiadzin Cauc, 22 IV 1946, 6399, W. Eichler (2: MIZ); Jerevan město, Razdan, 26–27.5.1988, J. Strejček lgt., (1 male, 1 female: NMP); Iran, Lorestan, 1.1960 leg. A. Warchałowski (1: AJC); Iran, Khorramabad, 19-V-77, M. Rapilly leg. (1: MNHN).

##### Diagnosis.

*Coccidula litophiloides* is very distinctive among *Coccidula* species with large produced posterior pronotal angles, and a prosternal process without carinae (which are present in all remaining species). With its general body shape slightly widening posteriorly and pronotum distinctly widened laterally with broad lateral bead appearing glabrous, it is similar to *C. scutellata*. Male genitalia are distinctive with large, elliptical parameres possessing projections on their inner surfaces, which is also unique among *Coccidula*. Spermatheca, in female genitalia, is distinctly widening apically and has a very short sperm duct, about ¼ of the length of spermatheca.

##### Description.

Length 3.0–3.5 mm, BL/BW = 1.95–1.97, EL/BW = 1.32–1.40, PW/BW = 0.81.

Body elongate, slightly widening in posterior part. Dorsal and ventral side yellow to testaceous (Fig. 1E).

Head and pronotum covered with uniform small setiferous punctures arranged irregularly. Pronotum transverse, broadly rounded laterally (Figs 1E, 3B), with broad, glabrous lateral margin, covered with dense setriferous punctures, with a single row of larger punctures along lateral border. Posterior pronotal corner large, distinctly pointed (Fig. 3B). Prosternum with complete anterior bordering line. Prosternal process without lateral carinae (Fig. 3C).

Scutellar shield pentagonal, covered with dense setiferous punctures. Elytra covered with two types of punctures, small setiferous punctures irregularly distributed throughout elytral surface, some of these punctures surrounded by larger depressed circles, forming irregular longitudinal rows; rows 2 and 3 reduced or missing (Fig. 3A). Elytra more flattened in lateral view than in other *Coccidula*, without shoulder tubercle, lateral elytral margin visible throughout (Fig. 3A). Hind wings missing. Mesoventrite with anterior border complete. Metaventrite with postcoxal lines transverse, descending only laterally, fused on metaventral process in median part, forming continuous arc (Fig. 3D); covered with setiferous punctures very sparsely distributed in central part of sclerite, densely setose in lateral parts, without distinct rows of large punctures below postcoxal lines, large punctures above metacoxae present.

Abdominal postcoxal lines complete, rounded, reaching slightly less than half of length of the ventrite 1 measured below metacoxa. Ventrites covered with dense setiferous punctures.

***Male genitalia*.** Tegmen in inner view with penis guide pentagonal with pointed apex (Fig. 4B); short, slightly longer than half length of parameres (Fig. 4A). Parameres large, elliptical, inner surface with distinct projections (Fig. 4B), with fringe of long setae in apical margin. Penis simple with pointed apex (Fig. 4C).

***Female genitalia*.** Sperm duct short (Fig. 4D), about as long as 1/4 of spermatheca. Spermatheca vermiform, distinctly broadened apically. Accessory gland membranous, longer than sperm duct.

##### Type locality.

Caucasus, Ordubad (Azerbaijan).

##### Distribution.

Armenia, Azerbaijan, Iran

##### Remarks.

Duverger (1983) described *Lithophilus naviauxi* from Iran. After examination of the type specimens (Fig. 1A, B) we noticed that this species does not belong to the genus *Lithophilus* Frölich (=*Tetrabrachys* Kapur). As drawn in the original publication (Duverger 1983), it has antennae with 11 antennomeres (10 in *Tetrabrachys*), and pseudotrimerous tarsi with tarsomere 3 very small and tarsomere 2 distinctly lobed, while in *Tetrabrachys* tarsi are distinctly tetramerous, with tarsomere 3 and 2 elongate, without distinct lobe. Duverger in his paper (1983) described *L. naviauxi* based on just three female specimens of which he illustrated the spermatheca (Duverger 1983: 89, figs 30, 31). However, *C. litophiloides* is also found in Iran. Comparison of the female genitalia of the type material of both taxa, and other available material, together with the lack of a second and third row of large punctures on the elytra, and other morphological features described in the original description of Duverger, led to the conclusion that *L. naviauxi* Duverger falls well within the definition of *C. litophiloides*; thus, we propose to synonymize both species.

#### Coccidula reitteri

Taxon classificationAnimaliaColeopteraCoccinellidae

Dodge, 1938

D80F83A5-0C5E-5F05-A570-C7F38CA8AB88

**Figure 6.**
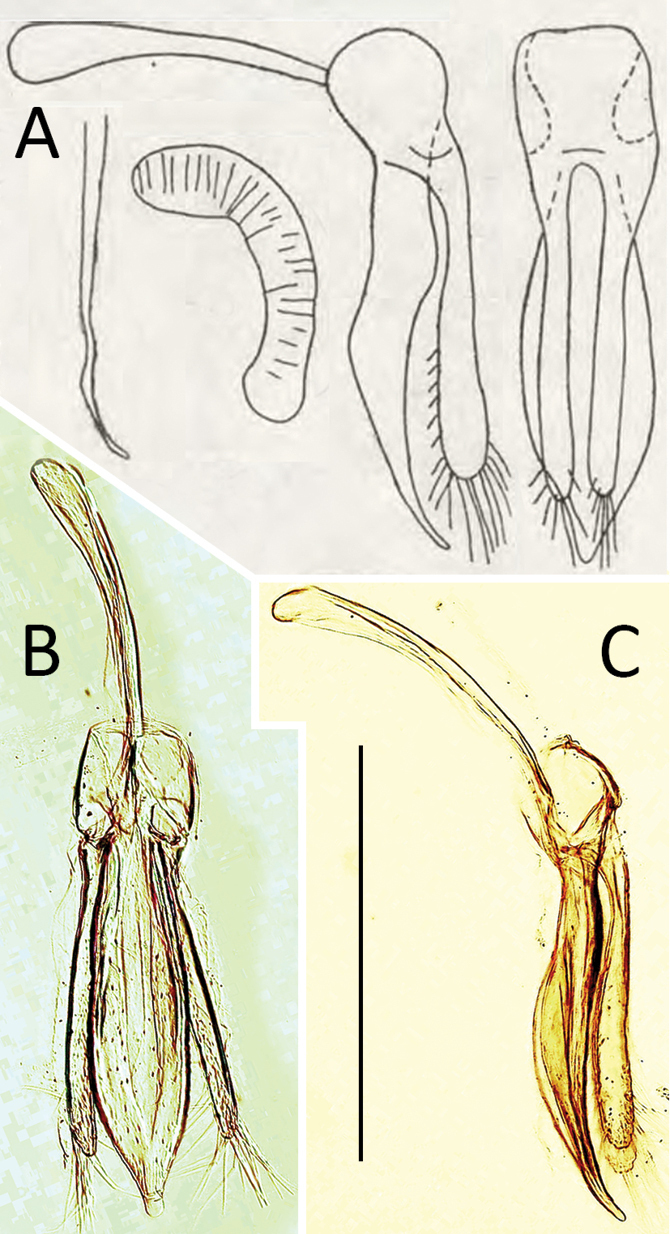
*Coccidula reitteri* Dodge **A** original drawings of male and female genitalia by Bielawski 1984**B** tegmen, inner **C** tegmen, lateral. Scale bar: 500 µm (**B, C**).

Figs 1F , 5A–E , 6A–C

Coccidula suturalis Reitter 1897: 127 nom. nud. (nec. C. suturalis Weise, 1895: 132).
Coccidula reitteri Dodge, 1938: 222.

##### Material examined.

***Holotype*.** Russia, “Quell. d. Jrbut Reitter./ Transbaikal leg. Leder/ Coll. Reitter/ / Coccidula scutellaris m 1896/ Coccidula reitteri Dodge Khnzorian det./ prep. genital R. Bielawski 1956/ Holotypus 1897 Coccidula suturalis Reitter/ Photo ID: HNHM_COL_574”, female (HNHM). **Other material.** Russia, “Transbaikalien Leder Reitter/ Coccidula suturalis Rtt. Coll. Reitter/ Coccidula reitteri Dodge, det. Merkl 1984/ prep. genital R. Bielawski 1956” (1 male: HNHM); Listvjanka pr. Bajkal, step, 29.6.1977, H. Karnecka lgt. (1 male, 1 female: NMP).

##### Diagnosis.

*Coccidula reitteri* is very similar to *C. rufa* in external appearance, however, it can be distinguished by the presence of a small black transverse macula on the pronotum just anterior to the scutellar shield, and a longitudinal brown to black macula on the posterior half of the elytra on the elytral suture. Male genitalia are very close to *C. rufa*, however, the upper margin of the penis guide in lateral view is relatively less emarginated and parameres are narrower than in *C. rufa*.

##### Description.

Length = 2.8–3.2 mm, BL/BW = 1.85–1.90, EL/BW = 1.33, PW/BW = 0.77.

Body elongate, parallel sided (Fig. 5A). Pronotum (Fig. 1F) with black transverse macula in front of the scutellar shield. Scutellar shield black. Elytra brown with elongate, dark brown to black macula along the elytral suture in posterior half. Ventral side testaceous with prosternum, mesoventrite, metaventrite, most of the ventrite 1 (except lateral corners), and central part of ventrite 2 black.

Head and pronotum covered with uniform small setiferous punctures arranged irregularly. Pronotum (Fig. 5B) transverse, broadly rounded laterally, with moderately broad, lateral margin without glabrous area; pronotum covered with dense setriferous punctures, with single row of larger punctures along lateral border. Posterior pronotal corners not produced (Fig. 5B). Prosternum with anterior margin with bordering line incomplete in median part, without small sub-rounded impression in center. Prosternal process with lateral carinae straight, joined together roundly at level of anterior border of procoxae, forming sub-triangular pattern (Fig. 5C).

Scutellar shield pentagonal, covered with dense setiferous punctures. Elytra covered with two types of punctures, small setiferous punctures irregularly distributed throughout the elytral surface, some of these punctures surrounded by larger depressed circles forming nine irregular longitudinal rows along whole length of elytra. Lateral elytral margin well visible throughout (Fig. 5A). Mesoventrite with complete anterior border. Metaventrite with postcoxal lines descending laterally, fused on metaventral process in median part, forming continuous arc (Fig. 5D); covered with setiferous punctures very sparsely distributed in central part of sclerite, densely setose in lateral parts, without distinct rows of large punctures below postcoxal lines, large punctures above metacoxae present.

Abdominal postcoxal lines complete, arcuate, reaching half of length of ventrite 1 measured below metacoxa. Ventrites covered with sparse setiferous punctures.

***Male genitalia*.** Tegmen in inner view (Fig. 6B) with penis guide broadly rounded in the median or apical part, with rounded apex; in lateral view (Fig. 6C) moderately expanded medially, with upper surface moderately emarginate; long, much longer than parameres. Parameres elongate, parallel sided, with narrow base, inner surface smooth, with fringe of long setae in apical part. Penis simple with pointed apex, with small bump before apex.

***Female genitalia*.** Spermatheca vermiform, not distinctly broadened apically (Fig. 6A).

##### Type locality.

Mongolia, Russia (Krasnoyarsk region, Irkutsk region, Tuva)

##### Distribution.

Russia (East Siberia).

##### Remarks.

*Coccidula reitteri* is very similar to *C. rufa* in external morphological characters as well as the structure of male and female genitalia (Fig. 6A) (Bielawski 1984); thus, further investigation, preferably of molecular markers, should be conducted to confirm whether it is a separate species or an eastern population of *C. rufa*.

#### Coccidula rufa

Taxon classificationAnimaliaColeopteraCoccinellidae

(Herbst, 1783)

25B90092-038B-55CE-8660-9DF331C51381

**Figure 7.**
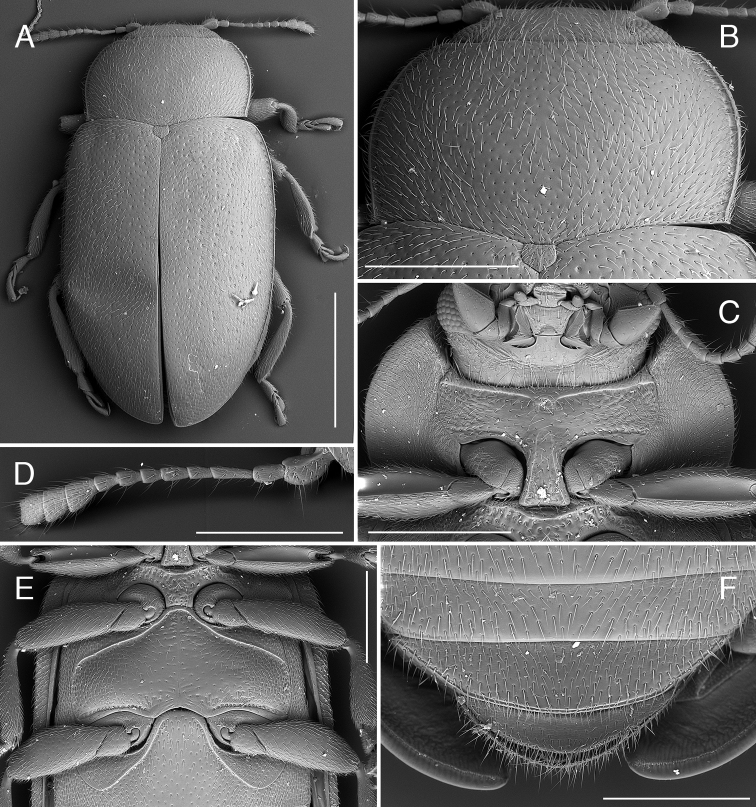
*Coccidula rufa* (Reitter) SEM illustrations **A** body, dorsal **B** pronotum **C** head and prothorax, ventral **D** antenna **E** mesoventrite, metaventrite and ventrite 1 **F** ventrites 4–6, female. Scale bars: 1 mm (**A**); 500 µm (**B, C, E, F**); 400 µm (**D**).

**Figure 8.**
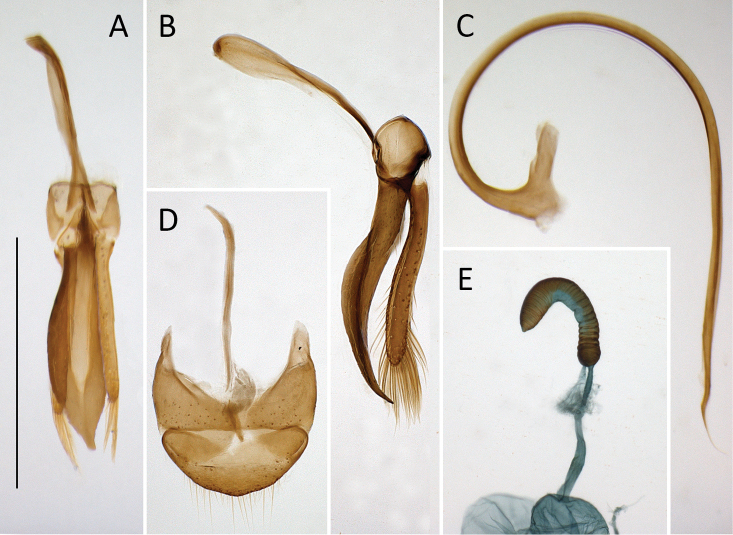
*Coccidula rufa* (Reitter) **A** tegmen, inner **B** tegmen, lateral **C** penis, lateral **D** male genital segment, dorsal **E** spermatheca. Scale bar: 500 µm (**A–E**).

Figs 1A, B, G , 7A–F , 8A–E

Dermestes rufus Herbst, 1783: 22.
Chrysomela pectoralis Fabricius, 1792: 328.
Silpha rosea Marscham, 1802: 123.
Coccidula conferta Reitter, 1890: 176.
Coccidula rufa var. unicolor Reitter, 1890: 176.
Coccidula rufa var. nigropunctata Reitter, 1900: 220.
Coccidula rufa var. plagiata Gerhardt, 1910: 556.

##### Material examined.

Czech Rep., Zlín, 11.6.1999, lgt. L. Bureš (1: NMP); Mladá Boleslav, 25.4.1987, lgt. Nedvěd (1 male USB); Dvořiště, 9.8.1989, lgt. Nedvěd (1: USB); Kokořínský důl, 9.8.1995, lgt. J. Řehounek (1: USB); Kyrgyzstan, Toktogul, 26 VI 2003, leg. A. Lasoń, WJ 2870, (1 male: AJC); Montenegro, Skadar jez.- Virpazar, 5.6.1984, J. Strejček lgt. (1: NMP); Poland, Kampinos Forest near Warsaw, 17.06.2020, leg. D. Marczak, (7: MIZ); Russia, Leningrad-Lachta, IX 1988, J. Strejček lgt., (1: NMP); Ukraine, Kharkiv region, Dergachevsky district, Boliboki vill., 50°9'16.57"N, 36°3'58.87"E, 1.5.2017, lgt. A. Slutsky (1: ASC); Uzbekistan, Buchara/ Coccidula unicolor Rtt./ Coll. Reitter/ MIZ PAN Warszawa 27/1955/1 (1: MIZ). Type material not studied, deposited in Museum für Naturkunde, Berlin, Germany.

##### Diagnosis.

*Coccidula rufa* is most similar in external appearance to *C. reitteri*, however it can be separated by the uniform testaceous coloration of the dorsal surface (*C. reitteri* possesses dark macula near the elytral suture). From uniformly colored specimens of *C. scutellata* it can be separated by the shape of carinae on the prosternal process. Male genitalia are also very distinctive: in *C. scutellata* penis guide is small, about half length of parameres, while in *C. rufa* it is longer than parameres. Spermatheca in female genitalia of *C. rufa* is vermiform, not widening apically, while in *C. scutellata* it is distinctly widened in apical part.

##### Description.

Length = 2.5–3.2 mm, BL/BW = 1.88–2.00, EL/BW = 1.38–1.44, PW/BW = 0.80–0.82.

Body elongate, parallel sided. Elytra of typical (European) form testaceous without maculae (Fig. 1G), only scutellar shield dark brown to black. Ventral side testaceous with prosternal process, mesoventrite, metaventrite, most of the ventrite 1 (except lateral corners), and central part of ventrite 2 black.

Head and pronotum covered with uniform small setiferous punctures arranged irregularly. Pronotum transverse, broadly rounded laterally, with moderately broad, lateral margin without glabrous area (Fig. 7B); pronotum covered with dense setiferous punctures, with a single row of larger punctures along lateral border. Posterior pronotal corners not produced (Fig. 7B). Prosternum with anterior margin with incomplete bordering line in median part, with a small sub-rounded impression in center. Prosternal process with lateral carinae straight, joined together roundly at level of anterior border of procoxae, forming sub-triangular pattern (Fig. 7C).

Scutellar shield pentagonal, covered with dense setiferous punctures. Elytra covered with two types of punctures, small setiferous punctures irregularly distributed throughout elytral surface, some of these punctures surrounded by larger depressed circles forming nine irregular longitudinal rows along whole length of elytra. Lateral elytral margin well visible throughout (Fig. 7A). Mesoventrite with complete anterior border. Metaventrite with postcoxal lines descending laterally, fused on metaventral process in median part, forming continuous arc (Fig. 7E), covered with setiferous punctures very sparsely distributed in central part of sclerite, densely setose in lateral parts, without distinct rows of large punctures below postcoxal lines, large punctures above metacoxae present.

Abdominal postcoxal lines complete, arcuate, reaching half of length of ventrite 1 measured below metacoxa. Ventrites covered with sparse setiferous punctures.

***Male genitalia*.** Tegmen in inner view (Fig. 8A) with penis guide sub-parallel to broadly rounded, with rounded apex; in lateral view (Fig. 8B) expanded medially, with deeply emarginated upper margin; long, much longer than parameres. Parameres elongate, parallel sided, with just slightly narrower base, inner surface smooth, with fringe of long setae in apical part. Penis simple with sharply pointed and curved apex, with small bump before apex (Fig. 8C).

***Female genitalia*.** Sperm duct long, longer than spermatheca (Fig. 8E). Spermatheca vermiform, not distinctly broadened apically. Accessory gland membranous, much shorter than sperm duct.

##### Type locality.

Berlin (Germany)

##### Distribution.

Europe (all countries), Africa: Morocco, Asia: Afghanistan, China, Russia (Siberia), Iran, Kazakhstan, Kyrgyzstan, Mongolia, Turkey, Uzbekistan.

#### Coccidula scutellata

Taxon classificationAnimaliaColeopteraCoccinellidae

(Herbst, 1783)

69A8F176-F3EC-52D7-83FC-33DB64C28042

**Figure 9.**
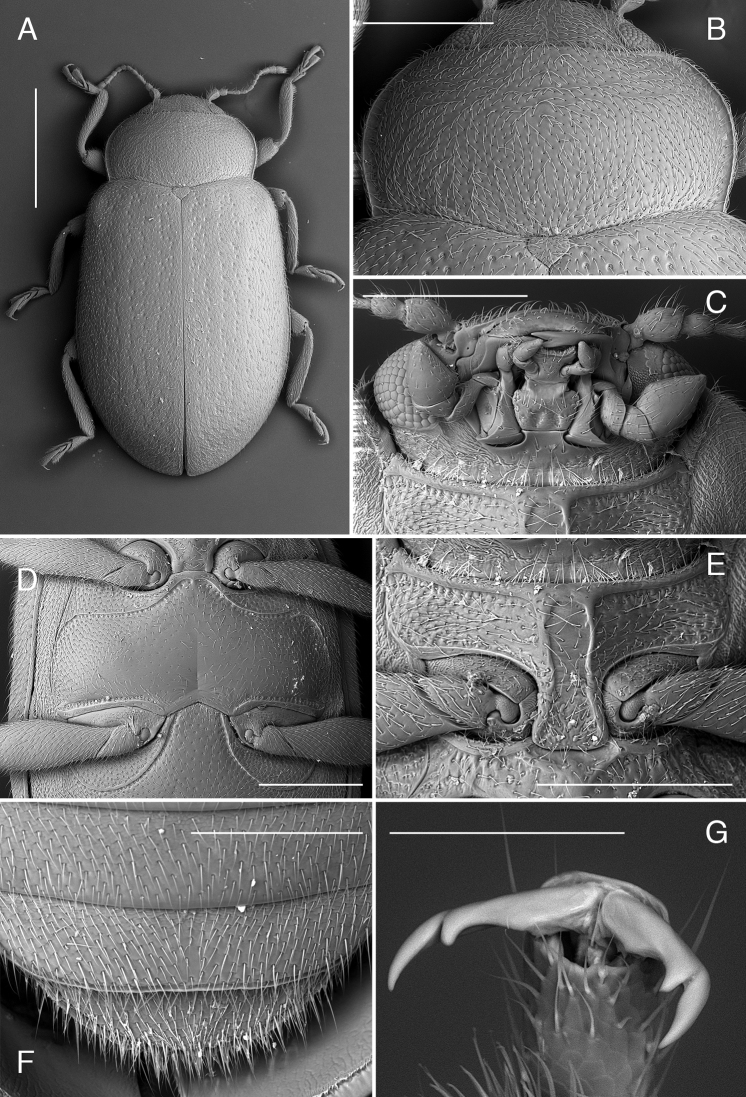
*Coccidula scutellata* (Reitter) SEM illustrations **A** body, dorsal **B** pronotum **C** head, ventral **D** mesoventrite, metaventrite and ventrite 1 **E** prosternum **F** ventrites 4–6, male **G** pro-tarsal claw. Scale bars: 1 mm (**A**); 500 µm (**B, D, E**); 400 µm (**C, F**); 100 µm (**G**).

**Figure 10.**
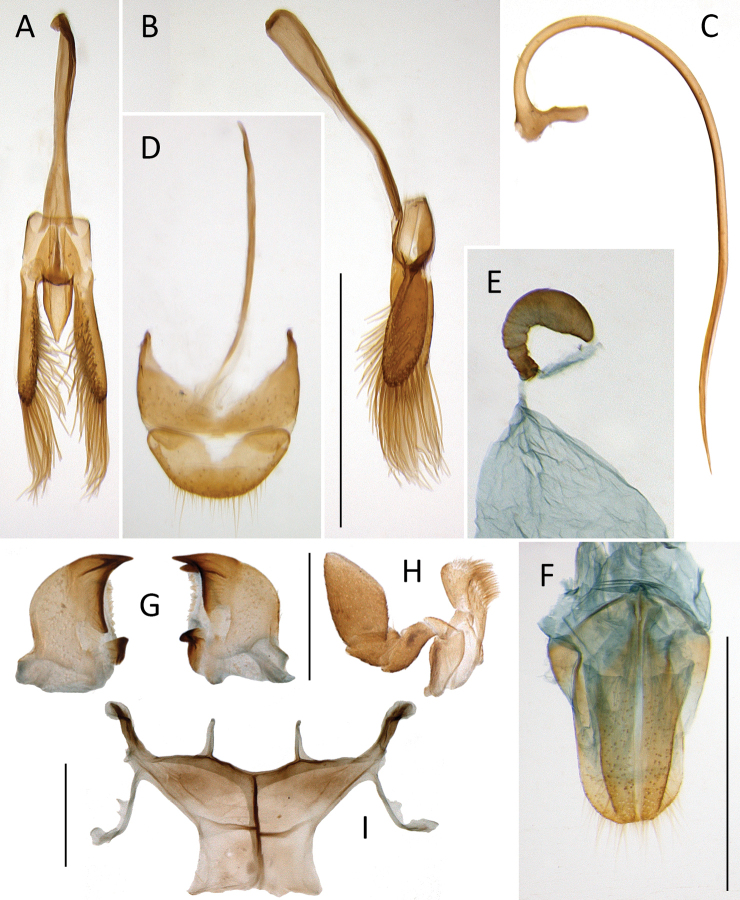
*Coccidula scutellata* (Reitter) **A** tegmen, inner **B** tegmen, lateral **C** penis, lateral **D** male genital segment, dorsal **E** spermatheca **F** female genitalia **G** left and right mandibles **H** maxilla **I** metendosternite. Scale bars: 500 µm (**A–F**); 200 µm (**G–I**).

Figs 1H , 9A–G , 10A–I

Chrysomela scutellata Herbst, 1783: 58.
Nitidula quinquepunctata Fabricius, 1787: 52.
Silpha melanophthalma Gmelin, 1790: 1627.
Nitidula bipunctata Gmelin, 1790: 1630
Coccidula scutellata : Kugelann 1798: 421.
Coccidula scutellata var. subrufa Weise, 1879: 131.
Coccidula scutellata var. arquata Weise, 1879: 131.
Coccidula scutellata var. aethiops Krauss, 1902: 92.

##### Material examined.

Armenia, Erevan, 9.06.1987, leg. V. Karasjov (5: AJC); Czech Rep., Praha-Kyje, 21.1.1945, lgt. Günnther, (1: NMP); Plzeň, 20.7.1978, lgt. V. Mach, (2: USB); Kokořínský důl, 28.8.1994, lgt. J. Řehounek (1: USB); Loučeň, 17.8.1994, lgt. J. Řehounek (1: USB); France, St. Cucufa, VI 65, MD, Ch. ‘Duverger det., J.P. Coutanceau det. 2004’ (1: MNHN); Poland, Kampinos Forest near Warsaw, 17.06.2020, leg. D. Marczak (11: MIZ); Slovakia, Bratislava, 27.4.36, lgt. O. Kavan (1: NMP); Ukraine, Kharkiv region, Kharkiv district, Bobrovka vill., reserve “Aleshkina balka”, 2017-04-28, lgt. A. Slutsky (1: ASC). Type material not studied, deposited in Museum für Naturkunde, Berlin, Germany.

##### Diagnosis.

*Coccidula scutellata* is the most variable species in body coloration. Typical forms with five black maculae on the elytra can be easily distinguished from other *Coccidula* species, however uniformly colored testaceous forms are externally similar to *C. rufa*. They can be easily distinguished by the shape of carinae on prosternal process, which are straight and form a sub-triangular pattern in *C. rufa*, and are sinuate and broadly rounded apically, and fused with anterior border of prosternum in *C. scutellata*. Moreover, *C. scutellata* has a more distinct shoulder tubercle, and relatively narrower protnotum. Also, the male genitalia are distinctive, with penis guide longer than parameres in *C. rufa* and much shorter in *C. scutellata*. Spermatheca, in female genitalia, is broadened apically in *C. scutellata*, while in *C. rufa* it is almost uniform in diameter.

##### Description.

Length = 2.8–4.2 mm, BL/BW = 1.85–2.05, EL/BW = 1.36–1.46, PW/BW = 0.70–0.75.

Body elongate, slightly widening in posterior part. Elytra of typical (European) form testaceous with five black maculae (Fig. 1H), one large covering scutellar shield and surrounding portion of elytra, and four sub-oval maculae in the median part, two of which are placed close to elytral suture and remaining two, close to lateral margin. Sometimes macula surrounding scutellar shield extends along elytral suture, sometimes maculae placed in median part of elytra are fused, forming single band. Various forms with reductions of this pattern are also present to completely testaceous forms without any trace of black color. Ventral side testaceous with prosternal process, mesoventrite, metaventrite, most of ventrite 1 (except lateral corners), and central part of ventrite 2 black.

Head and pronotum covered with uniform small setiferous punctures arranged irregularly. Pronotum transverse, broadly rounded laterally, with broad, glabrous lateral margin (Fig. 9B); pronotum covered with dense setiferous punctures, with single row of larger punctures along lateral border. Posterior pronotal corners not produced. Prosternum with anterior margin with bordering line complete. Prosternal process with complete lateral carinae in form of sinuate line, joined roundly and merged with anterior border of pronotum (Fig. 9E).

Scutellar shield pentagonal, covered with dense setiferous punctures. Elytra covered with two types of punctures, small setiferous punctures irregularly distributed throughout elytral surface, some of these punctures surrounded by larger depressed circles forming nine irregular longitudinal rows along whole length of elytra. Shoulder tubercles distinct, lateral elytral margin of elytra not visible from above in anterior part (Fig. 9A). Mesoventrite with anterior border interrupted in median part. Metaventrite with postcoxal lines transverse in median part and then descending laterally, not fused on metaventral process in median part (Fig. 9D). Covered with setiferous punctures very sparsely distributed in central part of sclerite, densely setose in lateral parts, with single row of large punctures below postcoxal lines and above metacoxae.

Abdominal postcoxal lines complete, rounded, reaching slightly more than half of length of ventrite 1 measured below metacoxa. Ventrites covered with dense setiferous punctures.

***Male genitalia*.** Tegmen in inner view (Fig. 10A) with penis guide subtriangular with pointed apex; short, about two times shorter than parameres. Parameres elongate elliptical (Fig. 10B), inner surface smooth, with long setae on inner surface and in apical margin. Penis simple with pointed apex (Fig. 10C).

***Female genitalia*.** Sperm duct short, about as long as half of length of spermatheca (Fig. 10E). Spermatheca vermiform, distinctly broadened apically. Accessory gland membranous, longer than sperm duct.

##### Type locality.

Pomerania (Germany, Poland)

##### Distribution.

Europe (all countries), Africa: Morocco, Asia: Kazakhstan, Russia (West Siberia).

**Figure 11.**
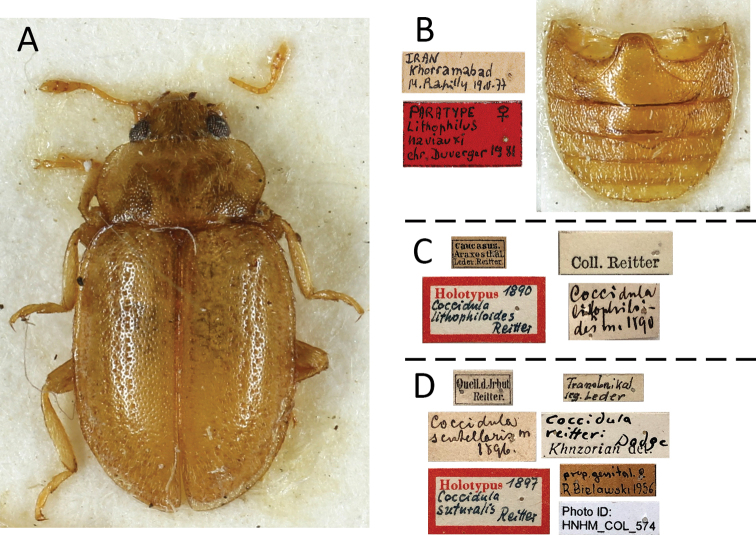
*Lithophilus naviauxi* Duverger **A** paratype MNHN, dorsal **B** paratype labels and abdomen **C***Coccidula litophiloides* Reitter, holotype labels **D***Coccidula suturalis* Reitter, holotype labels.

## Supplementary Material

XML Treatment for Coccidula

XML Treatment for Coccidula lepida

XML Treatment for Coccidula litophiloides

XML Treatment for Coccidula reitteri

XML Treatment for Coccidula rufa

XML Treatment for Coccidula scutellata
